# Wound-Induced Polyploidization: Regulation by Hippo and JNK Signaling and Conservation in Mammals

**DOI:** 10.1371/journal.pone.0151251

**Published:** 2016-03-09

**Authors:** Vicki P. Losick, Albert S. Jun, Allan C. Spradling

**Affiliations:** 1 Department of Embryology, Carnegie Institution for Science, Howard Hughes Medical Institute, 3250 San Martin Dr., Baltimore, MD 21218, United States of America; 2 Wilmer Eye Institute, Johns Hopkins School of Medicine, 400 N. Broadway, Baltimore, MD 21231, United States of America; University of Massachusetts Medical School, UNITED STATES

## Abstract

Tissue integrity and homeostasis often rely on the proliferation of stem cells or differentiated cells to replace lost, aged, or damaged cells. Recently, we described an alternative source of cell replacement- the expansion of resident, non-dividing diploid cells by wound-induced polyploidization (WIP). Here we show that the magnitude of WIP is proportional to the extent of cell loss using a new semi-automated assay with single cell resolution. Hippo and JNK signaling regulate WIP; unexpectedly however, JNK signaling through AP-1 limits rather than stimulates the level of Yki activation and polyploidization in the Drosophila epidermis. We found that polyploidization also quantitatively compensates for cell loss in a mammalian tissue, mouse corneal endothelium, where increased cell death occurs with age in a mouse model of Fuchs Endothelial Corneal Dystrophy (FECD). Our results suggest that WIP is an evolutionarily conserved homeostatic mechanism that maintains the size and synthetic capacity of adult tissues.

## Introduction

During the life of an organism physiological insults including injury, aging, and degenerative disease can damage tissues leading to the loss of mature cells. Consequently, a variety of mechanisms exist to compensate for cell loss and maintain adult tissue homeostasis [1–5]. One source of new cells is the division of resident tissue stem cells, which are present in many tissues including the skin, gut, and skeletal muscle [3, 6]. Differentiated cells provide a second source of new cells in tissues such as liver, where they can be induced to divide and contribute to cell replacement [7]. Animals that can regenerate their limbs also re-activate mature tissue cells to dedifferentiate to form the blastema and proliferate to compensate for cell loss [1, 8].

Recently a third source of cell replacement termed wound-induced polyploidization (WIP) was described in the adult Drosophila epidermis and hindgut [4, 9]. Post-mitotic differentiated diploid cells in these tissues respond to wounding by re-entering the cell cycle, but rather than proliferating mitotically they enter the endocycle and polyploidize. Polyploid cells have three or more copies of the haploid genome and are proportionately larger than diploid cells [5, 10]. Consequently, even though the total number of cells declines following wounding, the size and synthetic capacity of the tissue (i.e. the number of genomes per unit area) is maintained [4, 9].

Polyploid cells arise normally during development in diverse animals and plants by switching from the mitotic cycle to the endocycle [5, 10]. During the endocycle, the cell cycle resets without cytokinesis, generating a single large cell whose gene and cytoplasmic content (and hence its functional capacity) is equivalent to the two cells that would have resulted from division. Interestingly, animals bearing mutations that reduce cell proliferation may still develop organs of normal size made up of fewer, yet larger cells by causing cells to polyploidize. Such compensatory developmental polyploidization has been observed in Drosophila imaginal discs, ovarian follicular epithelium, and blood brain barrier [11–13], and may also occur in mammalian liver [14–16]. Whether the same signals are used to re-establish tissue homeostasis during development and in adult WIP remains unknown.

The Hippo and JNK signaling pathways play a critical role in controlling organ size during development [17–19]. In developing imaginal discs, knocking down *yorkie* (*yki*), blocks cell division resulting in reduced tissue growth. Conversely, enhancing Yki activity by overexpression or knocking down the upstream inhibitory kinase genes *hippo* or *warts* enhances cell division resulting in tissue overgrowth. JNK signaling adds another layer of Hippo-dependent growth control. This stress-induced conserved MAPK pathway has been observed to either promote or inhibit growth. During Drosophila development or intestinal stem cell-mediated regeneration, JNK signaling activates Yki and promotes cell proliferation and tissue growth [17, 20, 21]. However, studies of imaginal disc tumors showed that JNK signaling can either activate or inhibit Yki depending on the tissue context [22].

Here we systematically investigated several questions surrounding WIP using a semi-automated method that displays a tissue’s nuclear spatial distribution and ploidy values (DNA content) with single cell resolution. We find that the Drosophila epidermis tightly controls the spatial distribution, extent, and the magnitude of WIP such that the number of new genomes produced regionally closely matches the number of genomes lost to wounding. Moreover, Hippo and JNK signaling are not only necessary, but quantitatively control both the spatial extent and magnitude of polyploidization. Finally, we show that polyploidization is used in a mammalian tissue, the corneal endothelium, to compensate for cell loss during aging and disease. Our observations strengthen the view that WIP is an evolutionarily conserved homeostatic mechanism to compensate for cell loss that serves to maintain the size and functional capacity of adult organs.

## Results

### A new semi-automated assay reveals the extent and symmetry of wound-induced polyploidization

**Fig 1:**
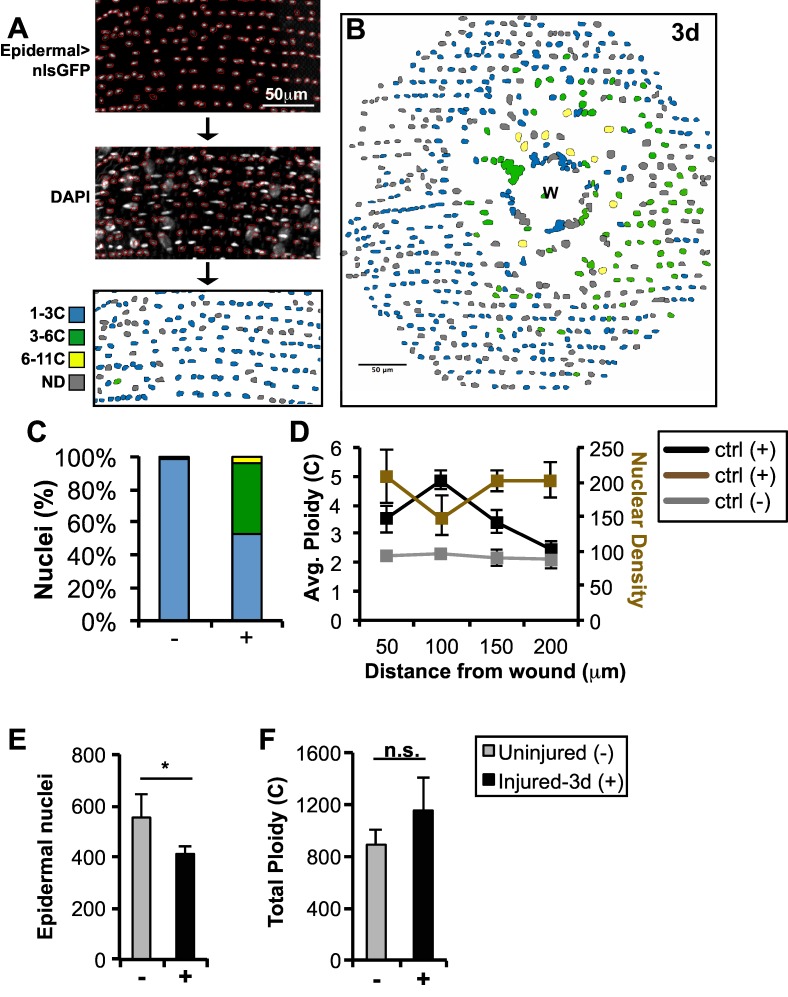
Wound-induced polyploidization compensates for cell loss in adult Drosophila epidermis. Schematic of the semi-automated approach to identify and measure DNA content within fly epidermis. Fly epidermal nuclei were identified by epidermal specific expression of nuclear GFP. Nuclei were outlined (red) using Fiji’s trace function and transferred to the corresponding DAPI image. Nuclear traces were color-coded based on the normalized ploidy value as indicated. Nuclei overlapping with non-epidermal nuclei were not determined (ND, gray). Epidermal cells polyploidize in response to injury: (A) Uninjured (-), (B) 3d post injury (+), and (C) % of epidermal nuclei in the indicated color-coded ploidy ranges. (D) Average epidermal ploidy (black or gray lines for injured (+) or uninjured (-), respectively) and nuclear density (brown line) versus distance from the wound center or image center in ctrl. (E) Epidermal nuclear number is reduced 3d post injury. (F) Total epidermal ploidy is not significantly different (n.s.) at 3d post injury. All analysis was preformed with 2 uninjured and 5 injured flies. Error bars represent standard deviation where *p<0.05 is based on two-tailed Student's *t* test.

We developed a new semi-automated method to count and determine the ploidy of epidermal nuclei within the Drosophila adult abdomen (Fig 1A; see Methods). In short, individual fly epidermal nuclei expressing a nuclear GFP transgene driven by the epidermal-Gal4, which is specific to the adult epidermis and co-localizes with epidermal nuclear marker Grainy-head (Grh), were identified using Fiji software and their outlines recorded (Fig 1 and S1 Fig) [23]. The total DAPI signal within each nuclear boundary was then measured and normalized to an internal control, fly spermatids. Nuclei that overlapped with non-epidermal nuclei could not be determined. This method provides a detailed picture of the distribution, size, and ploidy of most nuclei throughout the uninjured and repaired abdominal epidermal tissue 3 days (d) post injury (Fig 1A and 1B).

We used this approach to examine circular tissue regions of radius 150μm (about 71,000μm^2^) centered on the wounds three days post injury. Prior to wounding, no epidermal nuclei scored higher than 5.5c, and only 3% scored as tetraploid or G2 (3.5c to 6.4c). Three days after a puncture wound to the abdomen, wound healing is complete and epidermal cells have finished polyploidization [9]. Now, within the same region around the wound center, 42% of nuclei on average are tetraploid, 5% are octoploid (Fig 1B and 1C, color code), and the average ploidy has risen from 2.21 to 3.56.

This new analysis revealed a non-random pattern of polyploid nuclei (Fig 1B and 1D). The wound site itself remains covered by a scab under which lie few if any nuclei. At the edge of the scab is a dense clustering of nuclei that are part of a giant syncytial cell that has re-epithelialized the wound site under the scab [9]. Interestingly, many of the induced polyploid nuclei reside in the next 50μm or so outside the scab-covered region (50–100μm from the wound center). Consistent with this, the average nuclear ploidy rises to a peak of nearly 5c at 50μm -100μm from the wound, and falls back to diploid levels by about 150μm from the wound (Fig 1D). This 8c polyploid-rich 50–100μm region likewise is lower in total nuclei (Fig 1D), as expected if polyploidization acts to equalize DNA content regionally within the epidermis.

Many cells located 50–100μm from the wound probably migrate to the damage site and fuse to make the central syncytium shortly after wounding. The remaining nuclei in the cell-depleted zone then polyploidize to compensate for the lost cells. Plotting the DNA ploidy (S2A Fig) reveals that in controls the vast majority of the nuclei are diploid. After wounding, about 50% of nuclei retain a 2c DNA content, while the remainder have polyploidized, generating a peak around 4c and a tail of values up to 16c.

These new results argue that WIP is tightly regulated to compensate for cell loss. To investigate this further, we quantified the change in nuclear number and ploidy within this large (71,000μm^2^) region. There are 554 ±90 nuclei per region prior to wounding, but only 412 ±27 nuclei after wounding, showing that 142 diploid cells are lost on average (Fig 1E). Not every epidermal nucleus could be scored for ploidy due to random overlaps with non-epidermal nuclei in the fly abdominal tissue. However, 79% of uninjured nuclei and 67% of injured nuclei were measured, allowing an accurate estimate of the total epidermal ploidy. Summing the nuclear epidermal ploidy values within these regions reveals that 890 ±118 genomes prior to wounding are replaced by 1154 ±252 genomes following polyploidization (Fig 1F). These findings support our conclusion that WIP is a tightly controlled repair process that homeostatically maintains total tissue ploidy.

### The Hippo pathway controls the extent of endoreplication post injury

The Hippo pathway is known to control tissue growth by regulating cell cycling and survival. Our previous studies indicated that the Hippo pathway also regulates tissue growth by controlling entry into the endocycle [9]. We hypothesized that Yki may also tune the extent of polyploidization that occurs post injury. To address this hypothesis, Yki activity was genetically manipulated in the adult fly epidermis using the previously characterized epidermal-Gal4 driver [9].

**Fig 2:**
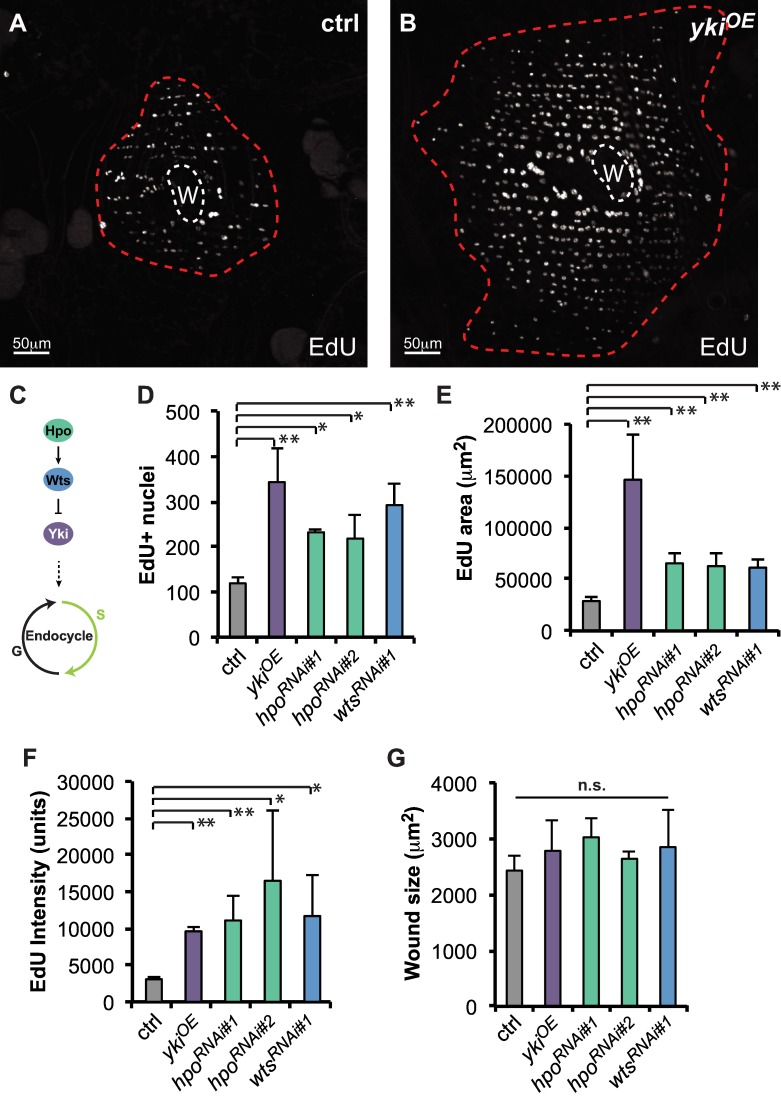
Hippo pathway controls the extent of endoreplication post injury. EdU marks endoreplicating cells around the wound scar (W, white dashed line) at 2d post injury. Immunofluorescent images of EdU staining in (A) ctrl or (B) epidermal specific Yki overexpression (*yki*^*OE*^). The boundary of EdU+ area is outlined (red dashed line). (C) Diagram of core Hippo signaling pathway regulating entry into the endocycle. (D-F) Quantification of the effect of core Hippo genes on EdU response where (D) represents the average number EdU+ nuclei, (E) the average area containing EdU+ cells, (F) average EdU intensity per nucleus, and (G) the wound scar size at 2d post injury. All constructs were expressed with epidermal specific-Gal4 driver and examined at 2d post injury. At least 3 flies were scored for each condition. Error bars represent standard deviation where *p<0.05, **p<0.01, and n.s., not significant (*p*>0.05) are based on two-tailed Student's *t* test.

In wild-type (ctrl) flies, a standard puncture wound to the adult abdomen induced cells around the wound to re-enter S phase and polyploidize as indicated by incorporation of the thymidine analog, EdU (Fig 2A). To determine if altered Hippo signaling affects this response, we upregulated Yki activity in the adult epidermis by overexpressing wild-type *yki* (epidermal-GAL4::UAS-*yki*^*OE*^) [24]. As expected under these conditions Yki was strongly induced in the adult epidermis (S3A Fig). Alternatively, the upstream negative regulatory genes *hippo* (*hpo*) or *warts* (*wts*): *hpo*^*RNAi*^ (epidermal-GAL4::UAS-*hpo*^*RNAi*^) or *wts*^*RNAi*^ (epidermal-GAL4::UAS-*wts*^*RNAi*^) were also knocked down (Fig 2C). In the absence of wounding, Yki overexpression did not change the appearance of abdominal epidermal tissue or EdU incorporation (see below). In contrast, after wounding Yki upregulation caused more nuclei to incorporate EdU throughout a larger area (Fig 2B, 2D and 2E). The level of EdU incorporation also increased 2–3 fold compared to wounded ctrl animals (Fig 2F). These results show that many more epidermal cells polyploidize in response to wounding and suggest that they undergo more endocycles when Yki activity is increased.

### The Hippo pathway controls tissue homeostasis by regulating endoreplication post injury

**Fig 3:**
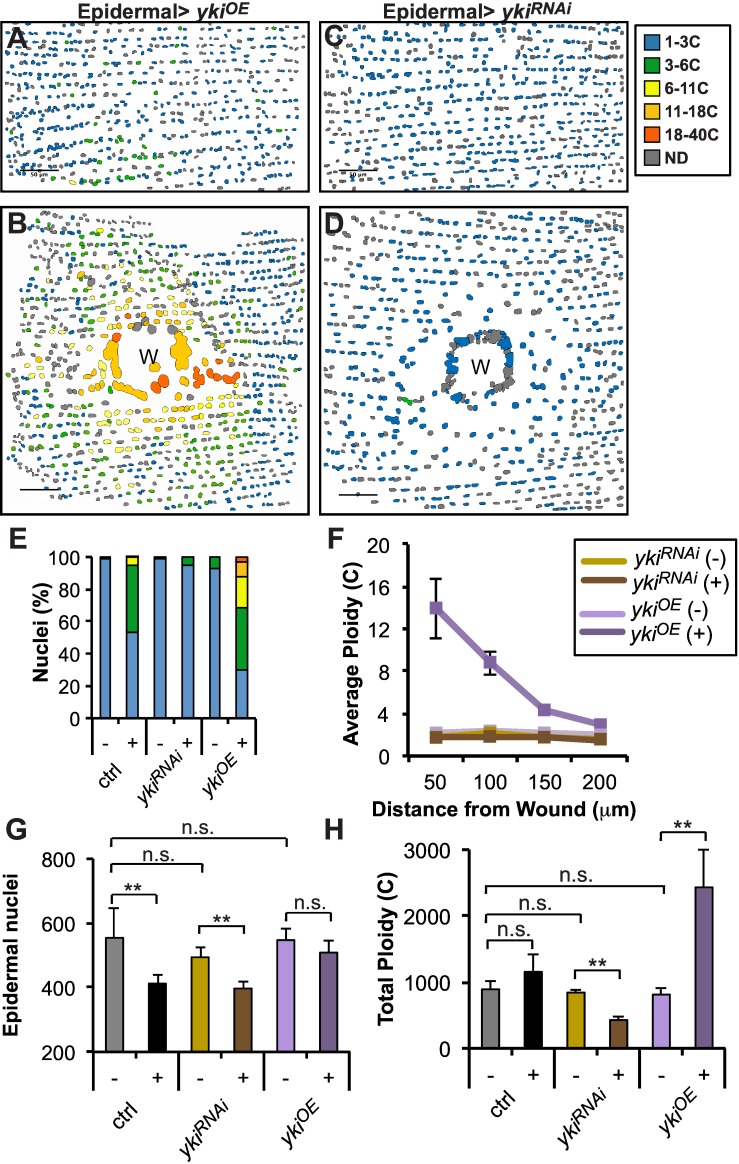
Yki tunes the extent of polyploization post injury. Spatial distrubution and ploidy values (as indicated by color code) of fly abdominal epidermal nuclei expressing *yki*^*OE*^ or *yki*^*RNAi*^ (A and C) Uninjured and (B and D) 3d post injury, respectively. Nuclei not determined (ND, gray). Scale bar, 50μm. (E) Ploidy (%) distribution in uninjured (-) and 3d post injury (+) in indicate fly genotypes. (F) Average epidermal ploidy and nuclear density versus distance from the wound center or image center in ctrl in the indicated conditions. (G) Epidermal nuclear number significantly declines 3 days post injury, except when *yki* is overexpressed in fly epidermis. (H) Total tissue ploidy is altered by *yki* expression. (E-H) All analysis was preformed with at least 2 uninjured and 3 injured flies for each condition. Error bars represent standard deviation where **p<0.01 and n.s., not significant (p>0.05) are based on two-tailed Student's *t* test.

We determined the spatial profile and extent of polyploidization under conditions where Yki activity is upregulated to further quantitate these effects (Fig 3). Consistent with the EdU experiments above, uninjured adult epidermal cells from *yki*^*OE*^ flies remain diploid (Fig 3A and 3E) and do not ectopically re-enter the cell cycle. However after injury, 47% to 70% of cells in *yki*^*OE*^ animals polyploidize, a significant increase compared to wild type and their levels of ploidy reach values as high as 32c (Fig 3B and 3E). Interestingly, the spatial distribution of polyploid cells also changed in *yki*^*OE*^ animals. The highest ploidy values were now observed near the wound site (Fig 3F), instead of in the zone 50–100μm away (Fig 1D). Not only were many nuclei induced to polyploidize up to and beyond 16c, but the DNA content of nearly all cells increased in a broad peak between 2c and 4c (S2B Fig).

Yorkie overexpression resulted in other dramatic changes in the wound healing response. The number of nuclei in the abdomen lost to wounding sharply declined from 142 in control to only 39 in *yki*^*OE*^ flies on average (Fig 3G). Although a substantial segment of abdominal tissue is lost and replaced with a normal-sized scab (Fig 2G), more cells appear to survive the trauma of wounding in *yki*^*OE*^ flies, probably because Yki is known to induce anti-apoptotic genes, including *dIAP*, which was upregulated even prior to injury (S3 Fig) [24, 25]. While fewer nuclei cluster around the wound site, they are still able to form a syncytium that allows for re-epithelialization (data not shown). Interestingly, the epidermal nuclear rows characteristic of abdominal tissue were significantly less disturbed by the standard puncture wound when Yki was overexpressed (Figs 3B vs 1B). The entire tissue appears to have been less mechanically perturbed by the wound, and yet, the tissue has over responded. Indeed we found that wounded *yki*^*OE*^ flies have more than a 2-fold increase in their ploidy within the studied region (Fig 3H).

We also analyzed the effects of lowering Yki activity in abdomens to look for reciprocal effects. We confirmed our previous observation that *yki*^*RNAi*^ blocks polyploidization in injured epidermis (Fig 3D, 3E and S2 Fig) and as a result, with our new analysis, caused a 2-fold loss of total tissue ploidy in the vicinity of the wound post injury (Fig 3H). There was no change in the clustering of nuclei at the edge of the scab (Fig 3D) and nuclear density was still reduced around the wound (Fig 3F). Under laboratory conditions, the reduction in polyploidization caused by *yki*^*RNAi*^ is not lethal, but it is likely to be suboptimal under more stressful conditions. In conclusion, the Yki activity level is critical to tune the extent of polyploidy that occurs post injury to ensure that tissue homeostasis is restored.

### JNK signaling and AP-1 regulate the extent of endoreplication post injury

**Fig 4:**
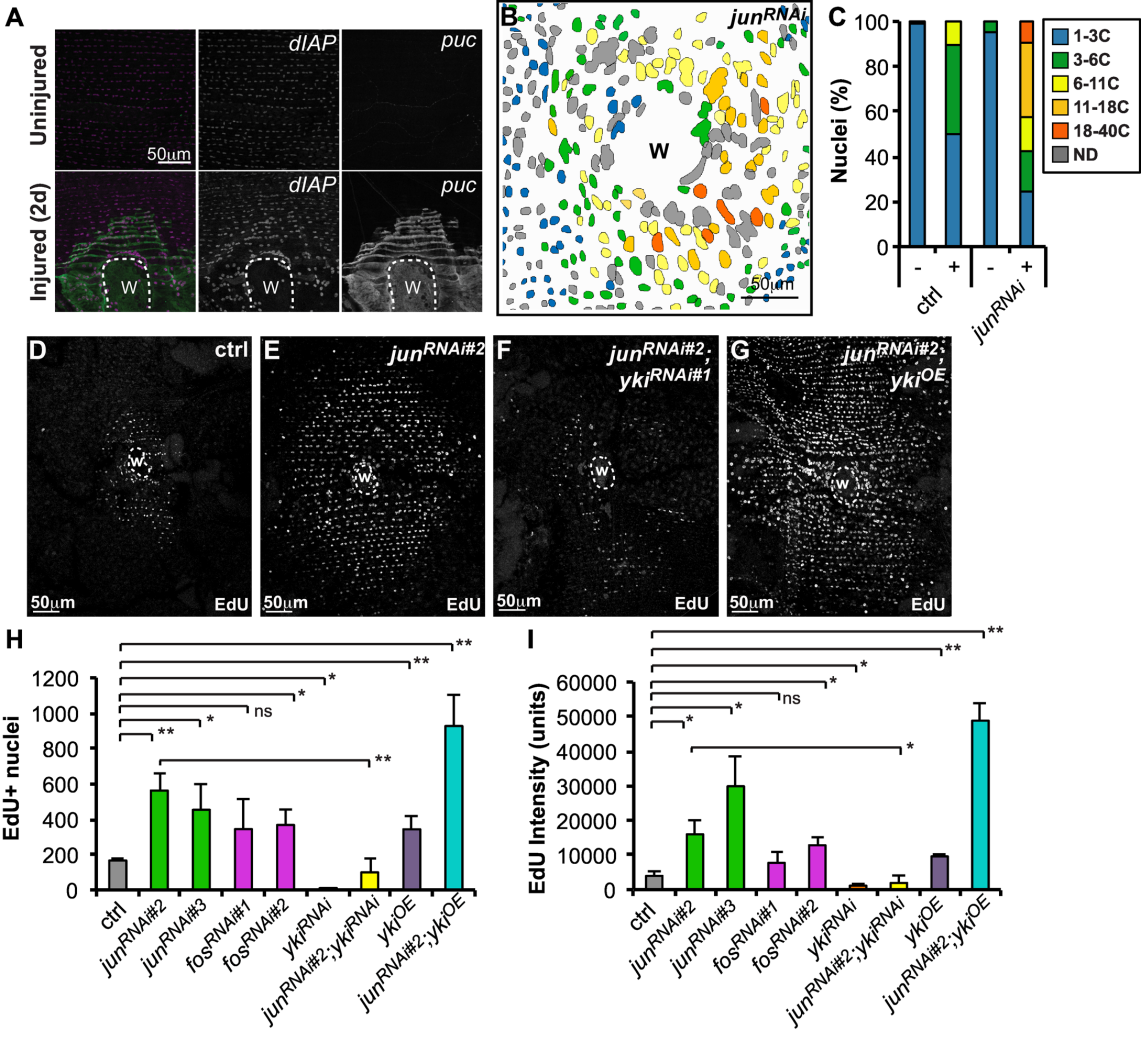
AP-1 regulates wound-induced polyploidy by affecting Yki. (A) Yki (*dIAP-lacZ*) and JNK (*puc*-Gal4, UAS-GFP) reporters are upregulated by injury and co-localize around wound site at 2d. (B) Spatial distribution and ploidy values (as indicated by color code) of fly abdominal epidermal nuclei expressing *jun*^*RNAi*^. Nuclei not determined (ND, gray). (C) Ploidy (%) distribution in uninjured (-) and 3d post injury (+) in indicated fly genotypes. Analysis was performed with 2 uninjured and 5 injured flies. (D-G) Immunofluorescent images of EdU stained fly abdomens at 2d post injury expressing indicated transgenes. (H and I) AP-1 transcription factors, *jun* and *fos*, affect endoreplication as measured by EdU incorportation. (I) is the average EdU intensity per nucleus. All transgenes were expressed with epidermal specific-Gal4 driver and examined at 2d post injury. At least 3 flies were scored for each condition. Error bars represent standard deviation where *p<0.05, **p<0.01, and n.s., not significant (p>0.05) are based on two-tailed Student's *t* test.

The JNK signaling pathway plays important roles in wound repair and is activated by a puncture wound in Drosophila [9, 26–28]. Several studies have indicated crosstalk between the Hippo and JNK signaling pathways [20–22]. Consequently, we investigated the role of JNK signaling in WIP and whether it interacts with Hippo signaling. The JNK reporter (*puc-lacZ*) was strongly induced by injury in epidermal cells surrounding the wound site (Fig 4A), and partially co-localized with cells upregulating the Yki reporter (*dIAP-lacZ*) [24, 29]. We knocked down several components of the JNK pathway using the epidermal-GAL4 driver to investigate whether JNK signaling affects the WIP response (S4 Fig). Knocking down either of the AP-1 transcription factor components, *jun* or *fos*, with multiple non-overlapping UAS-RNAi constructs consistently reduced or abolished JNK reporter expression at 2d post injury (S4 Fig).

We examined EdU incorporation and polyploidization surrounding the wound site to investigate whether reduced AP-1 activity influences the WIP response (Fig 4 and S4E–S4H Fig). Surprisingly, knocking down *jun* or *fos* had the opposite effect of knocking down *yki*. The number of EdU^+^ nuclei around the wound site and the strength of EdU incorporation increased 2–3 fold when either *jun*^*RNAi*^ or *fos*^*RNAi*^ were expressed in the epidermis (Fig 4D, 4E, 4H and 4I), although wound size was unchanged (S4H Fig). These effects required wounding, since genetically knocking down *jun* or *fos* did not detectably affect epidermal morphology or polyploidization (Fig 4C and data not shown). Thus, disrupting AP-1 function was similar to overexpressing wild-type Yki (Fig 2B).

We analyzed the epidermal ploidy from *jun*^*RNAi*^ flies and there was only a slight increase in polyploidy prior to injury, but substantially more polyploidization occurred following wounding compared to control flies (Fig 4B and 4C). Unlike *yki*^*OE*^, *jun*^*RNAi*^ flies had a strong disruption of the regular nuclear rows despite a similar increase and distribution of tissue ploidy as in *yki*^*OE*^ and showed fewer cells at the wound margin than wild type (Figs 1B vs 3B). In addition, a broad range of nuclear ploidy values resulted, rather than classes corresponding to a uniform polyploidization response (S2 Fig).

### AP-1 acts through Yorkie to modify wound-induced polyploidization

We inhibited JNK signaling in the adult epidermis and examined the expression of known Yki-dependent reporters post injury to determine if JNK signaling influences the activation of Yki during WIP [24]. Injury upregulates two Yki reporters, *dIAP-lacZ* and *ex-lacZ*, in epidermal cells surrounding the wound site (S5A, S5C and S5D Fig) [9], but in *jun*^*RNAi*^ animals, *dIAP-lacZ* and *ex-lacZ* expression were even further upregulated during the wound healing response (S5B–S5D Fig). We confirmed that *dIAP-lacZ* expression post injury is dependent on Yki (S3B Fig). In addition, knocking down or overexpressing *yki* in the fly epidermis does not affect expression of the JNK reporter, *puc-lacZ*, supporting our hypothesis that AP-1 functions to limit the activation of Yki during wound repair (S5E–S5I Fig).

We simultaneously knocked down both *yki* and *jun* (*jun*^*RNAi*^*; yki*^*RNAi*^) to test whether the *jun*^*RNAi*^ enhancement of polyploidization was due to Yki upregulation. Both the number and intensity of EdU-labeled nuclei near the wound site were strongly reduced in the double knockdown condition compared to *jun*^*RNAi*^ alone (Fig 4D–4F, 4H and 4I). In addition, polyploidization could be further enhanced by simultaneously knocking down *jun* and overexpressing *yki* (*jun*^*RNAi*^*; yki*^*OE*^) (Fig 4G, 4H and 4I), but wounding was still required. We concluded that the enhancement of wound-induced polyploidization caused by reducing the AP-1 transcription factors Jun and Fos was largely due to enhanced Yki activity in their absence.

### Enhanced polyploidization contributes to cellular homeostasis within the mouse corneal endothelium

Cell loss can be caused by variety of physiological insults, including injury, aging, or disease. It has been observed in several mammalian tissues that scattered cells grow in size, often referred to as cellular hypertrophy, after tissues experience stress conditions [4, 30]. However, the number of such cells, and their ploidy levels have not been investigated and compared to pre-stress levels. Based on our studies of Drosophila, we hypothesized that mammalian tissues also utilize wound-induced polyploidization as a homeostatic mechanism of tissue maintenance. To test this idea, we used mouse cornea endothelium, since endothelial cells are known to enlarge in several circumstances.

The cornea endothelium is the inner most layer of the cornea and is composed of a monolayer of differentiated post-mitotic endothelial cells similar to adult fly epidermis (S6A Fig) [31, 32]. In response to injury, aging, or the human genetic condition Fuchs Endothelial Corneal Dystrophy (FECD), endothelial cells die faster than they can be replenished [32, 33]. Some surviving cells enlarge and become more irregular in shape, and some become multinucleated, characteristics also observed in the Drosophila epidermis post injury [9]. The DNA content of enlarged endothelial cells in two human patients with corneal dystrophy were previously reported to be elevated, due to the frequent presence of multinucleated and/ or mono-nucleated polyploid cells [34]. We investigated whether these changes represent a WIP-like process, rather than a pathological response, as widely assumed.

**Fig 5:**
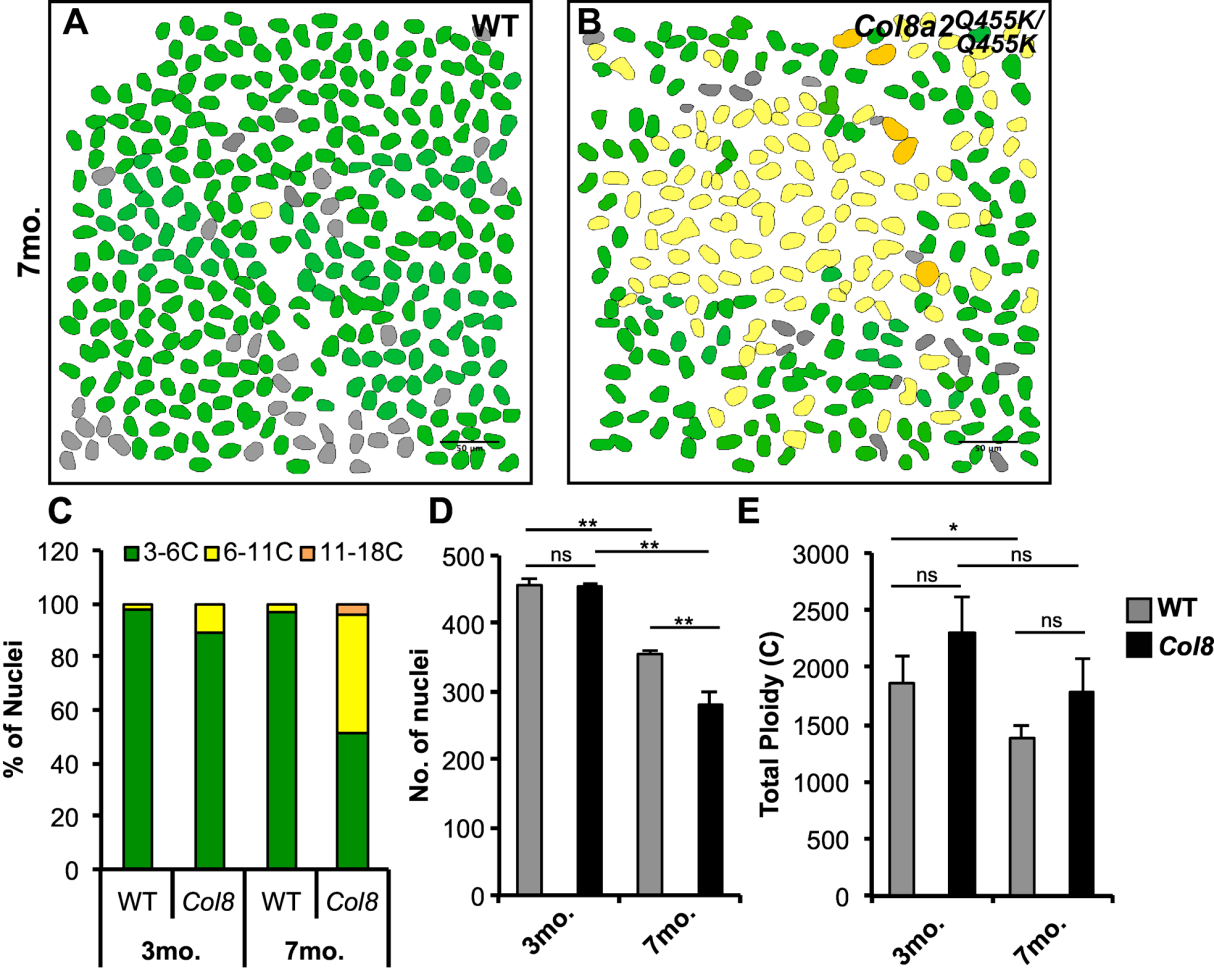
Mouse cornea endothelial cells increase ploidy to compensate for cell loss in Fuchs dystrophy mouse model. (A and B) Spatial distribution and ploidy values (as indicated by color code) of mouse endothelial nuclei from WT or *Col8a2*^*Q455K/Q455K*^ mice at 7 months old. Nuclei not determined (ND, gray). Scale bar, 50μm. (C) Ploidy (%) distribution in indicate mouse genotypes and ages. (D) Endothelial nuclear number significantly declines in *Col8a2*^*Q455K/Q455K*^ compared to WT mice by 7 months. (E) Total endothelial ploidy is not significantly different between WT and *Col8a2*^*Q455K/Q455K*^ mice. (C-E) Quantified from 3 representative endothelial (150,000μm^2^) regions within corneas from age matched male mice as represented in A and B. Error bars represent standard deviation where *p<0.05 and n.s., not significant (*p*>0.05) are based on two-tailed Student's *t* test.

We measured the DNA contents of mouse cornea endothelial cells in wild-type (WT) tissue and from seven-month old mice bearing the *Col8a2*^*Q455K/ Q455K*^ mutation [35]. This mutation accelerates the loss of endothelial cells while those remaining cells contain multiple and/ or enlarged nuclei, similar to the human endothelial layer in patients with FECD (S6 Fig) [34]. Diploid cornea epithelial nuclei were used as an internal control to quantitate the ploidy of both WT and *Col8a2*^*Q455K/ Q455K*^ endothelial nuclei. Our measurements showed that most normal corneal endothelial cells are at least twice as large and contain twice the DNA content of epithelial nuclei, indicating that they are tetraploid with a measured average ploidy of 4.6c (Fig 5A and S6C and S6D Fig). Interestingly, the corneal layer in *Col8a2*^*Q455K/ Q455K*^ mice contained many cells with even larger nuclei whose ploidy values ranged up to 16c and averaged 6.8c (Fig 5B and 5C and S6F and S6G Fig).

We compared these results to similar studies carried out at 3 months of age, to determine if polyploidization takes place over time to maintain the tissue ploidy levels in the mutant corneas (Fig 5D). WT and *Col8a2*^*Q455K/ Q455K*^ mice both have about 450± 11 endothelial cells in a test region of 150,000μm^2^ at 3 months of age. However, by 7 months, WT mice contained an average of 355± 6 endothelial nuclei whereas corneas from the *Col8a2*^*Q455K/Q455K*^ contained only 281± 19 endothelial nuclei within the same-sized region (Fig 5A, 5B and 5D) [35, 36]. The increased endothelial ploidy in the mutant maintained the total tissue ploidy (Fig 5E). Thus, *Col8a2*^*Q455K/Q455K*^ mutant mice contained 1,841c± 285c in the test region, similar to the total ploidy of 1647c± 106c in WT corneas. The close agreement of these values argues that polyploidization in response to endothelial cell loss, here caused by cell turnover in the *Col8a2*^*Q455K/Q455K*^ mutant mice, is a homeostatic rather than a pathological response that maintains the total biosynthetic capacity of the tissue. Unexpectedly, the wild type tissue did not undergo a similar WIP response, and its total ploidy was significantly lower at 7 months. The normal tissue may not have passed some threshold that depends on tissue disruption, in addition to just regional ploidy per se.

Next we asked whether there is evidence that the Hippo and JNK signaling pathway contribute to regulating polyploidization in FECD model. A sensitive method to measure signaling pathway regulation is to examine changes in gene expression. Gene expression changes were observed in cornea endothelial cells isolated from 12 month old *Col8a2*^*Q455K/Q455K*^ compared to WT mice [37], including known Hippo and JNK regulators. Two previously reported Yap target genes were upregulated: the G to S phase cyclin D, *ccnd2* (induced 4.26 fold, *p*-value = 0.0011) and the extracellular, matrix-associated signaling molecule, *cyr61* (induced 1.89 fold, *p*-value = 0.0091) [38–40]. Other upstream Yap-activating genes were also induced, including the E3 ubiquitin ligase *itch* (induced 2.60 fold, *p*-value = 0.0210) and the epidermal growth factor ligand, *nrg1* (induced 2.33 fold, *p*-value = 0.0006) [41, 42]. The AP-1 transcription factor *jun* was also reported to be upregulated 1.92 fold (*p =* 0.0001) in *Col8a2*^*Q455K/Q455K*^ compared to WT mice. *Jun* induction was confirmed and observed in patients with FECD [37]. While these changes were small, the polyploidization response may have occurred over a four-month period, rather than in just 2–3 days as in the case of an acute injury in Drosophila. All together these observations suggest that polyploidization in response to cell loss, is an adaptive tissue repair response and further genetic tests in mammals will determine if WIP also relies on the conserved signaling pathways, Hippo and JNK, to maintain adult tissue homeostasis.

## Discussion

### Wound-induced polyploidization occurs in mammals

We found that a mammalian tissue—the corneal endothelium, responds to cell loss in a nearly identical manner to the Drosophila abdominal epithelium [9]. Mouse corneal endothelial cells in animals bearing the *Col8a2*^*Q455K/Q455K*^ mutation are lost at an elevated rate as they age [35]. While the number of cells per unit area is similar in wild type and mutant animals at 3 months of age, by 7 months the mutants contained 35% fewer endothelial cells. In response, some of the previously quiescent cells, which are already tetraploid, re-enter the cell cycle and polyploidize further. DNA contents as high as 16c were observed, and the total amount of polyploidization was just sufficient to compensate for regional cell loss. These observations show that regulated polyploidization in adult mammals can compensate for cell loss and maintain tissue homeostasis. Pre-existing polyploid cells, like diploid cells, can respond to cell loss by re-entering the cell cycle and polyploidizing further.

Previous studies suggest that WIP may be occurring widely in other mammalian tissues. Scattered polyploid cells have been reported to arise in multiple tissues after physiological insults, including injury, aging, and degenerative disease [34, 43–47]. In the mammalian heart, following heart attack 60% of cardiomyocytes near the infarction site increase their ploidy from 4c to 8c [43]. Liver hepatocytes also polyploidize up to 64c following injury or as a consequence of aging [46–48]. Previously, these polyploid cells have been viewed as pathological [49–51]. We propose that the production of polyploid cells in these situations is beneficial and a manifestation of WIP [4].

### Wound induced-polyploidy is a conserved homeostatic response

Semi-automated ploidy analysis has expanded our knowledge of WIP. We obtained extensive evidence that polyploidization restores approximately the same number of genomes per unit area as in unwounded tissue, demonstrating that WIP is homeostatic. To achieve spatial and regional control, polyploidization must be tightly controlled and we found genetic conditions that perturb WIP and result in either an under or overcompensation of replacement genomes. These experiments also clarify the spatial distribution of induced polyploid cells. After wounding, fly epidermal cells become depleted 50–100μm from the wound center, likely due to the rapid migration of cells from this area toward the wound edge [9]. Preferential induction of polyploid cells in this depleted zone then acts to help restore a normal level of synthetic capacity to every zone within the epidermis.

#### The choice between proliferation and polyploidization

Why do some differentiated cells respond to wounding by proliferation and others by polyploidization? There is increasing evidence that the expression state of cell cycle genes such as mitotic cyclins in the responding cells is responsible [52]. Some tissue cells shut off mitotic genes as part of their differentiation program [53, 54]. The expression of mitotic genes is frequently controlled by the transcription factor Escargot (Esg), which is required for *cdc2* transcription [55]. In the Drosophila midgut, *esg* is expressed in stem cells, but turns off in differentiating daughter cells that polyploidize to become enterocytes [56, 57]. If a critical mitotic gene has become heterochromatic and cannot be induced, that cell might have no option but to polyploidize in response to stress.

Thus, the state of cell cycle gene expression in differentiated tissue cells, rather than the nature of the injury, may determine whether the tissue responds by proliferation or by polyploidization. Consistent with this, experimentally altered cell cycle programing can redirect the cellular response to wounding. Abdominal histoblasts will shift into an endocycle if the mitotic cyclin, *cdc2*, is removed genetically [55]. Moreover, in the developing fly trachea, ectopic expression of *cdc25* (as known as *string*) is sufficient to switch the progenitors in the reverse direction, from an endocycle to a mitotic cell cycle [58]. Thus, WIP may not differ very much in how it is induced from well-studied programs of proliferative cell repair. However, the unique advantages of growing large cells remain unexplored and are likely to be significant.

### Regulation of polyploidization by JNK and Hippo signaling

We found that two major pathways play important roles in controlling the extent of WIP. The conserved JNK signaling pathway is strongly induced in cells surrounding the wound [9, 28]. The pathway is turned on within 6 hours post injury [9], and this early activation of JNK probably mediates the cell migration needed to initiate wound repair [26, 27]. Consistent with this, epidermal nuclear clustering is reduced and nuclei remain more evenly dispersed when *jun* is knocked down (Fig 4B).

In addition, Hippo signaling is also strongly upregulated following wounding, and the level of Yki activation controls the spatial extent and magnitude of polyploidization. Recent studies in multiple systems have described a connection between Hippo signaling and the mechanical forces within a tissue [59]. When cytoskeletal tension is increased, Yki becomes more highly activated. The wounding protocol we used not only disrupts the epidermal layer directly, but also severs several of the underlying lateral muscle fibers [9]. This might shift some of the mechanical tension normally carried by these muscles to the epidermis, increasing its cytoskeletal tension and upregulating Yki. As repair proceeds, the restoration of mechanical equilibrium might down regulate Yki to normal levels and terminate the polyploidization response. Consistent with this, we observed that polyploidization was sometimes more extensive in the horizontal axis than along the vertical (anterior-posterior) axis (Fig 1B).

Contrary to our initial expectation, JNK signaling played a role in limiting rather than inducing Yki. Loss of JNK signaling via knockdown of the AP-1 transcription factors, *jun* and *fos*, causes hyper polyploidization similar to the effect of overexpressing Yki, and this effect was mostly Yki-dependent. This suggests JNK normally acts to limit the extent of Yki activation thereby helping to regulate the extent of polyploidization. The negative regulation is similar to recent findings from the fly imaginal disc tumor model demonstrating that depending on context JNK signaling can be either pro- or anti- growth [22, 60]. However, JNK has also been shown to positively regulate Yki activity in imaginal discs and the midgut [21, 61]. In both cases, JNK dependent Yki modulation acts through the upstream inhibitory kinase, Wts [21, 22]. It remains to be determined whether the same signaling circuitry exists in adult fly to mediate WIP.

## Conclusion

Our findings suggest that cellular damage caused by cell loss or wounding leads to alternative mechanisms of tissue repair depending on the cellular context: stem cell proliferation, differentiated cell proliferation, or differentiated cell polyploidization. Thus, the physiological state of the tissue’s resident cells strongly influences how the tissue responds and compensates for local tissue damage. In conclusion, we have further characterized wound-induced polyploidization and find that it is an essential, and widely used part of the metazoan healing arsenal.

## Materials and Methods

### Fly husbandry and strains

*Drosophila melanogaster* strains used in this study were reared on standard cornmeal agar yeast food at 25°C unless otherwise noted. The following fly were obtained as indicated for use in this study: R51F10-Gal4 (Epidermal-Gal4) [9], UAS-*yki*^*OE*^ [24], UAS-*yki*^*RNAi*^ (TRiP HMS00041, Bloomington# 34067), UAS-*hpo*^*RNAi#1*^(TRiP HM500006, Bloomington #33614), UAS-*hpo*^*RNAi#2*^ (TRiP JF02740, Bloomington #27661), UAS-*wts*^*RNAi#1*^(TRiP HMS00026, Bloomington #34064), *dIAP-lacZ* (P{w[+mC] = lacW}th[j5C8]/TM3, Sb[1], Bloomington #12093), *ex-lacZ* (P{w[+mC] = lacW}ex[k12913]/CyO, Bloomington #11067), *puc-Gal4*, *UAS-GFP* (Enrique Martin-Blanco, CSIC), *puc-lacZ* (P{ry[+t7.2] = lArB}puc[A251.1F3] ry[506]/TM3, Sb[1], Bloomington #11173), UAS-jnkDN (Bloomington #6409), UAS-*jnk*^RNAi#1^ (VDRC #34138), UAS-*jnk*^RNAi#2^ (VDRC #104569), UAS-*jun*^RNAi#2^(VDRC #107997), UAS-*jun*^*RNAi#3*^ (TRiP JF01184, Bloomington #31595), UAS-*fos*^RNAi#1^(TRiP JF02804, Bloomington #27722), and UAS-*fos*^RNAi#2^(VDRC #6212). Multiple RNAi lines were used which target distinct regions of the target gene to control for off target effects. R51F10-Gal4/+ was used as wild-type (ctrl) flies. Epidermal nuclei were labeled for ploidy analysis with high specificity by combining Epidermal-GAL4 with flpout nlsGFP [Ubip63E(FRT.STOP)Stinger9F6] and UAS-Flp as previously described [9].

### Mouse husbandry

Homozygous mutant knock-in mice harboring the *Col8a2*^Q455K/Q455K^ point mutation and homozygous *Col8a2* wild-type (WT) mice were generated by Dr. Albert Jun’s laboratory as previously described [35]. All mice were euthanized with isoflurane (Vedco Inc., St. Joseph, MO) verified by checking for the absence of respirations and followed by cervical dislocation. Four WT and four *Col8a2*^Q455K/Q455K^ mutant animals were used in this study. Animals were maintained and treated under specific pathogen-free conditions. All experiments were performed according to the ARVO Statement for the Use of Animals in Ophthalmic and Vision Research and adhering to protocols approved and monitored by the Animal Care and Use Committee of the Johns Hopkins University School of Medicine.

### Fly wound assay, dissection, and histology

Three to five days old adult female flies were punctured once on either side of the ventral midline between tergites A2 and A6 with a 0.10mm stainless steel insect pin (Small parts). At indicated times abdomens were dissected in Grace’s insect cell medium (Invitrogen) at room temperature under a light dissecting microscope. The internal organs were removed and the abdomens were filleted open by cutting along the dorsal midline with dissecting Vannas spring scissor (Fine Science Tools, #15000–00). Filleted abdomens were pinned down on a Sylgard plate with four 0.10mm insect pins.

Filleted abdomens were fixed in 3.7% formaldehyde in 1x PBS for 30 minutes at room temperature while pinned open to Sylgard plates. Primary antibodies were used overnight at 4°C. Antibodies and dilutions used in this study are rabbit anti-GFP (Invitrogen, 1:2000), mouse anti-FasIII (DSHB, 1:50), chicken anti-βgal (Abcam, preabsorbed,1:1000), rabbit anti-Yki [24], and rabbit anti-Grh [23]. Secondary antibodies from Invitrogen included donkey anti-rabbit 488, goat anti-rat 568, and goat anti-chicken 488 (1:500). Stained abdomens were mounted in vectashield (Invitrogen) on glass coverslip, with the inside tissue facing out. Images were taken on Leica Sp5 confocal microscope or ZEISS ApoTome and processed with Fiji/ ImageJ software to compile a maximum z-stack projection. Image brightness and contrast was linearly adjusted with Adobe Photoshop software.

### Wound size measurements

Wound scab was outlined and the area calculated using Fiji/ ImageJ software. At least three wounds were analyzed per conditions and average wound size and STDEV was calculated.

### EdU assay and quantification

Flies were fed 75μl of 5mM EdU yeast slurry 2 days prior to injury and continued until 2 days post injury. EdU was detected according to manufactures instructions (Click-it EdU Imaging Kit, Invitrogen). The number of EdU+ nuclei and EdU area was quantified from immunofluorescent images taken at 20x. The average EdU intensity ± STDEV was measured for epidermal nuclei within 100μm of the wound center. At least 50 nuclei were scored per image and at least 3 fly biological replicates were analyzed per condition.

### Semi-automated ploidy analysis

There is an extensive history successfully using DAPI fluorescence on tissue preparations as we do here to measure nuclear DNA content [62–64]. The accuracy of these methods have been extensively documented using the Drosophila ovary where there is natural polyploidy of nurse cells between 2c and 512c, and in follicle cells from 2c to 32c. The results are comparable to FACs profiles and can pick up DNA differences caused by under-replication of less than 0.1c.

For Drosophila, epidermal DNA ploidy was determined for a 71,000μm^2^ region surrounding each wound. Drosophila was prepared as described previously [9], except filleted abdomens were fixed and treated while pinned open. Samples were imaged using a Leica Sp5 confocal microscope with a 40×oil immersion objective and processed with Fiji software to compile a SUM of the z-stack projections. Fly spermatids were used as an internal control for ploidy measurements by imaging testis within each slide at same gain and settings. Using Fiji, regions were drawn around each nucleus based on the epidermal specific nuclear GFP expression using the trace function. RoiSet regions were recorded and transferred to the corresponding DAPI SUM of the z-stack image. The DAPI intensity was measured within each outlined nuclear region. The average background was calculated and subtracted from the measured intensities. The average DAPI intensity of sperm cells (1c) on same slide were analyzed and calculated. The epidermal ploidy was calculated by normalizing the epidermal DAPI intensity to average value of the 1c spermatids. The outlined nuclei were then color-coded based on their normalized ploidy value and binned into the indicated color-coded groups. Nuclei that overlapped with other non-epidermal nuclei in the abdominal tissue could not be determined and were colored gray (ND). Nuclei that clustered close together, particularly at wound edge had to be quantified as a group and then averaged. Based on this semi-automated approach nuclear number and tissue ploidy was calculated. **Nuclear number quantification:** Epidermal nuclei were identified by nls-GFP expression and the number was quantified by calculating the average nuclear number ± STDEV for a 71,000μm^2^ region per fly genotype for the indicated number of replicates. **Total Tissue Ploidy Quantification:** The ploidy value of all measurable nuclei was summed together for the 71,000μm^2^ region. The average total tissue ploidy was then calculated ± STDEV for each fly genotype for indicated number of replicates.

### Mouse cornea flatmounts, imaging, and ploidy analysis

WT and *Col8a2*^*Q455K*^/ ^*Q455K*^ mice were euthanized and the normal or Fuchs dystrophy phenotype was confirmed by clinical confocal microscopy (Nidek Confoscan3) [35]. Globes were enucleated using curved microsurgical forceps and scissors and placed in ice-cold PBS. Number 11 surgical blades were used to puncture the globe posterior to the limbus, followed by extension of the incision using curved microsurgical scissors to excise the corneas at the limbus. Attached iris tissue was removed using microsurgical forceps. Corneas were rinsed in ice-cold PBS and placed epithelial side down in the center of a Snowcoat X-tra glass slide (Surgipath). Number 11 surgical blades and microsurgical forceps were used to create 4 radial relaxing incisions 90° apart to allow the highly curved corneal tissue to lie flat on the slide. Excess fluid was wicked away using laboratory tissues, and corneas were then fixed for 30 minutes in a drop of 4% formaldehyde in PBS. During all manipulations, care was taken to minimize trauma to the corneal endothelial surface. Cornea samples were then permeabilized for 30 minutes in 0.3% Triton-X100 with 0.3% BSA. Primary rabbit ZO-1 antibody (1:200, Life Technologies) was diluted in same buffer and applied overnight. Secondary goat anti-rabbit 488 (1:500, Invitrogen) was diluted in permeablization buffer and applied for 3 hours. 4′,6-diamidino-2-phenylindole (DAPI, 1:1000, Invitrogen) was added as nuclear counterstain. Tissue was washed in 1xPBS and then mounted in vectashield (Invitrogen).

For mouse corneas, images were taken on Leica Sp5 confocal microscope using 20x objective. Images were processed similarly to Drosophila tissue using Fiji software and the semi-automated ploidy analysis. The only differences were that a larger 150,000μm^2^ region was sampled and epithelial cell nuclei were used as the internal control to calculate endothelial ploidy values. **Nuclear number quantification:** Endothelial nuclear number was quantified by calculating the average nuclear number ± STDEV of three microscopic visual fields of 150,000μm^2^ per cornea. **Total Tissue Ploidy Quantification:** The ploidy values of all measurable nuclei were summed together for the 150,000μm^2^ region. The average total tissue ploidy was then calculated ± STDEV for each mouse genotype with at least three replicates.

## Supporting Information

S1 Fig Epidermal-Gal4 allows for tissue specific expression in the fly epidermis. Epidermal-Gal4 driving expression of nlsGFP (Ep>nlsGFP) co-localizes with the epidermal marker Grainyhead (Grh, top panel) and does not co-localize with the lateral muscle fibers (middle panel). The fly epidermis forms a continuous sheet (cell-cell junctions are marked by FasIII) underlying the lateral abdominal muscle fibers.
(TIF)

S2 Fig Histogram plots of polyploidization within adult Drosophila epidermis. Peak ploidy values do not precisely correspond to a doubling of the fly genome, suggesting under or over-replication may occur during the wound-induced polyploidy response. Uninjured (-) and 3d post injury (+). Ploidy values were pooled from fly abdomens analyzed in (A) ctrl: (-) n = 2, (+) n = 5; (B) *yki*^*OE*^: (-) n = 2, (+) n = 3; (C) *yki*^*RNAi*^: (-) n = 1, (+) n = 3; (D) *jun*^*RNAi*^: (-) n = 2, (+) n = 3.
(TIF)

S3 Fig Yki regulation in adult Drosophila epidermis. (A) Robust *yki* overexpression or knockdown in adult fly epidermis. Immunofluorescent images of fly abdominal tissue in ctrl, *yki*^*RNAi*^, or *yki*^*OE*^. Yki activity was detected with *dIAP-lacZ* and Yki expression was detected with antibody generated against Yki. Row of epidermal nuclei marked by arrowhead. Other nuclei present in image are muscle nuclei. (B) Yki reporter expression, *dIAP-lacZ*, is dependent on *yki* post injury. Shown is 2d post injury. Wound scar (W, dashed white line).
(TIF)

S4 Fig Knocking down AP-1 transcription factor blocks JNK activation and affects wound-induced polyploidy. (A and B) Immunofluorescent images of the JNK reporter (*puc-lacZ*) expression around the fly wound at 2d post injury. (C) Diagram of core JNK signaling components. JNK (also known as Bsk in flies) activates the transcription factors Jun and Fos (making up the AP-1 complex) inducing Puc, a phosphatase, which feeds back to inhibit JNK. (D) Effectiveness of JNK pathway RNAi lines to inhibit the *puc-lacZ* expression. Shown is the average number of *puc-lacZ*^*+*^ nuclei at 2d. At least 10 flies were scored for each condition. (E and F) Immunofluorescent images of EdU staining in ctrl or epidermal specific *jun* knock down (*jun*^*RNAi*^). EdU area is outlined (red dashed line). Wound scar (W, dashed white line). (G and H) Quantification of the AP-1 genes’ effect on the EdU response and wound size at 2d post injury. All transgenes were expressed with epidermal specific-Gal4 driver and examined. At least 3 flies were scored for each condition. Error bars represent standard deviation where ** *p*<0.01, * p<0.05 and n.s., not significant (p>0.05) are based on two-tailed Student's *t* test.
(TIF)

S5 Fig AP-1 is upstream of Yki. Knocking down *jun* in the adult fly epidermis enhances Yki dependent gene expression. Shown are representative immunofluorescent images of (A) ctrl and (B) *jun*^*RNAi*^ for the Yki reporter *dIAP-lacZ* at 2d post injury. (C) Quantification of Yki reporters *dIAP-lacZ* and (D) *ex-lacZ* expression in uninjured (-) or at 2d post injury (2d) in indicated conditions. (E-H) Yki signaling does not affect activation of the JNK reporter. Shown are representative immunofluorescent images of (E) ctrl, (F) *jun*^*RNAi*^, (G) *yki*^*RNAi*^, and (H) *yki*^*OE*^ for the JNK reporter, *puc-lacZ*, at 2d post injury. (I) Quantification of JNK reporters in indicated conditions. Wound scar is outlined (W, dashed white line). At least 3 flies were scored for each condition. Error bars represent standard deviation where *p<0.05, **p<0.01, and n.s., not significant (*p*>0.05) are based on two-tailed Student's *t* test.
(TIF)

S6 Fig Mouse cornea endothelial cells are polyploid. (A) A cross-section diagram of the cell types within mammalian cornea of the eye. The outer most layer is made of several layers of epithelial cells (Ep, red) and the inner most layer is a single row of endothelial cells (En, blue) separated by a stromal cell layer (yellow). (B, C, E and F) Immunofluorescent DAPI images of the (B and C) WT and (E and F) *Col8a2*^*Q455K/Q455K*^ cornea Ep and En nuclei. Ep cells actively divide (arrowheads) and nuclei are small (example outlined) compared to differentiated En cell’s nuclei which are post-mitotic and larger. DNA content values were quantified from 3 representative endothelial (150,000μm^2^) regions from Ep vs En nuclei measured in (D) WT and (G) *Col8a2*^*Q455K/Q455K*^ within corneas from age matched 7-month old male mice.
(TIF)
