# Supplementary figures and images for: Wound-Induced Polyploidization: Regulation by Hippo and JNK Signaling and Conservation in Mammals
Source: PLoS One. 2016 Mar 9;11(3):e0151251. doi: 10.1371/journal.pone.0151251 (PMC4784922; doi:10.1371/journal.pone.0151251)

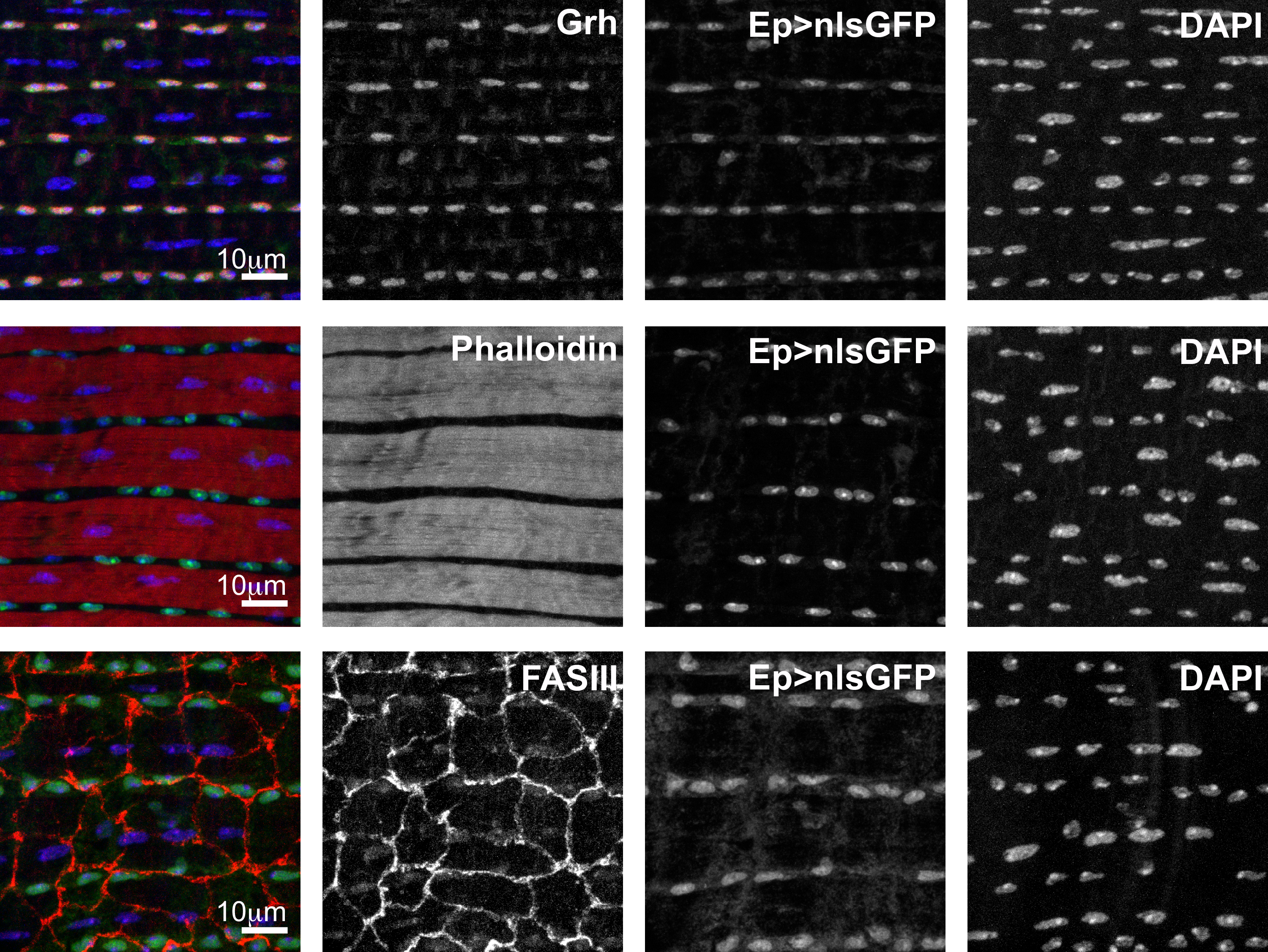

Supplement: S1 Fig — Epidermal-Gal4 driving expression of nlsGFP (Ep>nlsGFP) co-localizes with the epidermal marker Grainyhead (Grh, top panel) and does not co-localize with the lateral muscle fibers (middle panel). The fly epidermis forms a continuous sheet (cell-cell junctions are marked by FasIII) underlying the lateral abdominal muscle fibers. (TIF) [file pone.0151251.s001.tif]

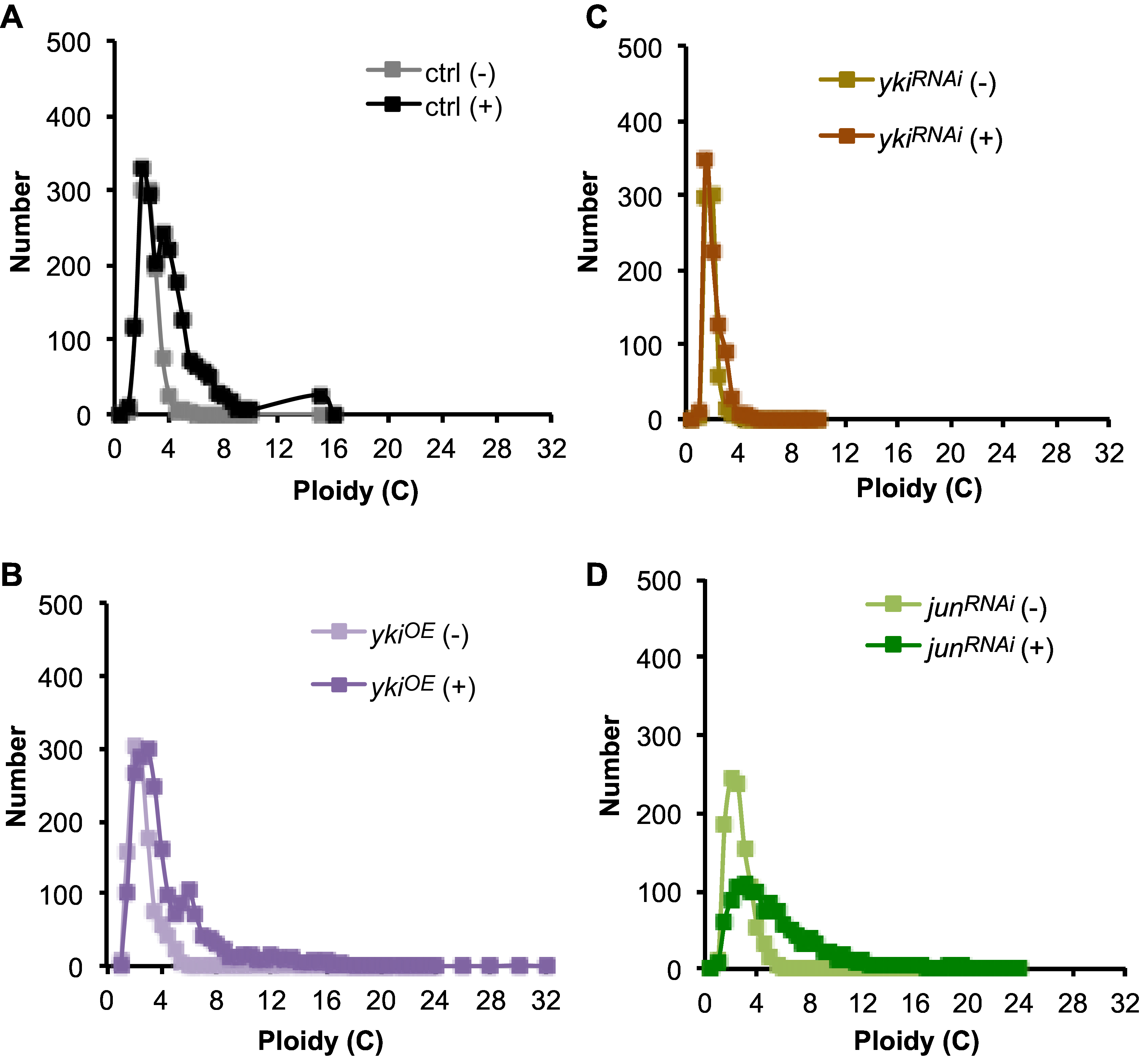

Supplement: S2 Fig — Peak ploidy values do not precisely correspond to a doubling of the fly genome, suggesting under or over-replication may occur during the wound-induced polyploidy response. Uninjured (-) and 3d post injury (+). Ploidy values were pooled from fly abdomens analyzed in (A) ctrl: (-) n = 2, (+) n = 5; (B) ykiOE: (-) n = 2, (+) n = 3; (C) ykiRNAi: (-) n = 1, (+) n = 3; (D) junRNAi: (-) n = 2, (+) n = 3. (TIF) [file pone.0151251.s002.tif]

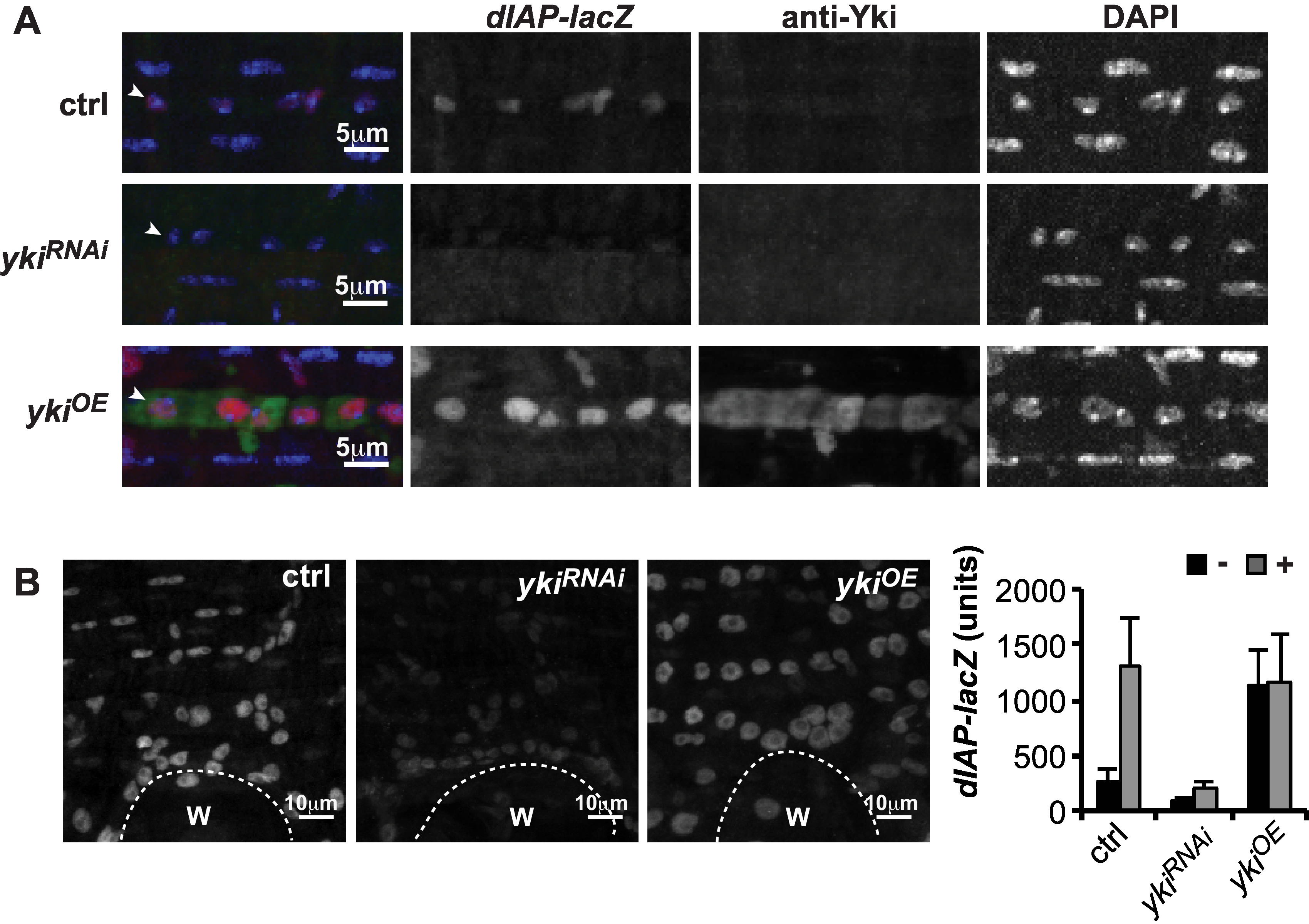

Supplement: S3 Fig — (A) Robust yki overexpression or knockdown in adult fly epidermis. Immunofluorescent images of fly abdominal tissue in ctrl, ykiRNAi, or ykiOE. Yki activity was detected with dIAP-lacZ and Yki expression was detected with antibody generated against Yki. Row of epidermal nuclei marked by arrowhead. Other nuclei present in image are muscle nuclei. (B) Yki reporter expression, dIAP-lacZ, is dependent on yki post injury. Shown is 2d post injury. Wound scar (W, dashed white line). (TIF) [file pone.0151251.s003.tif]

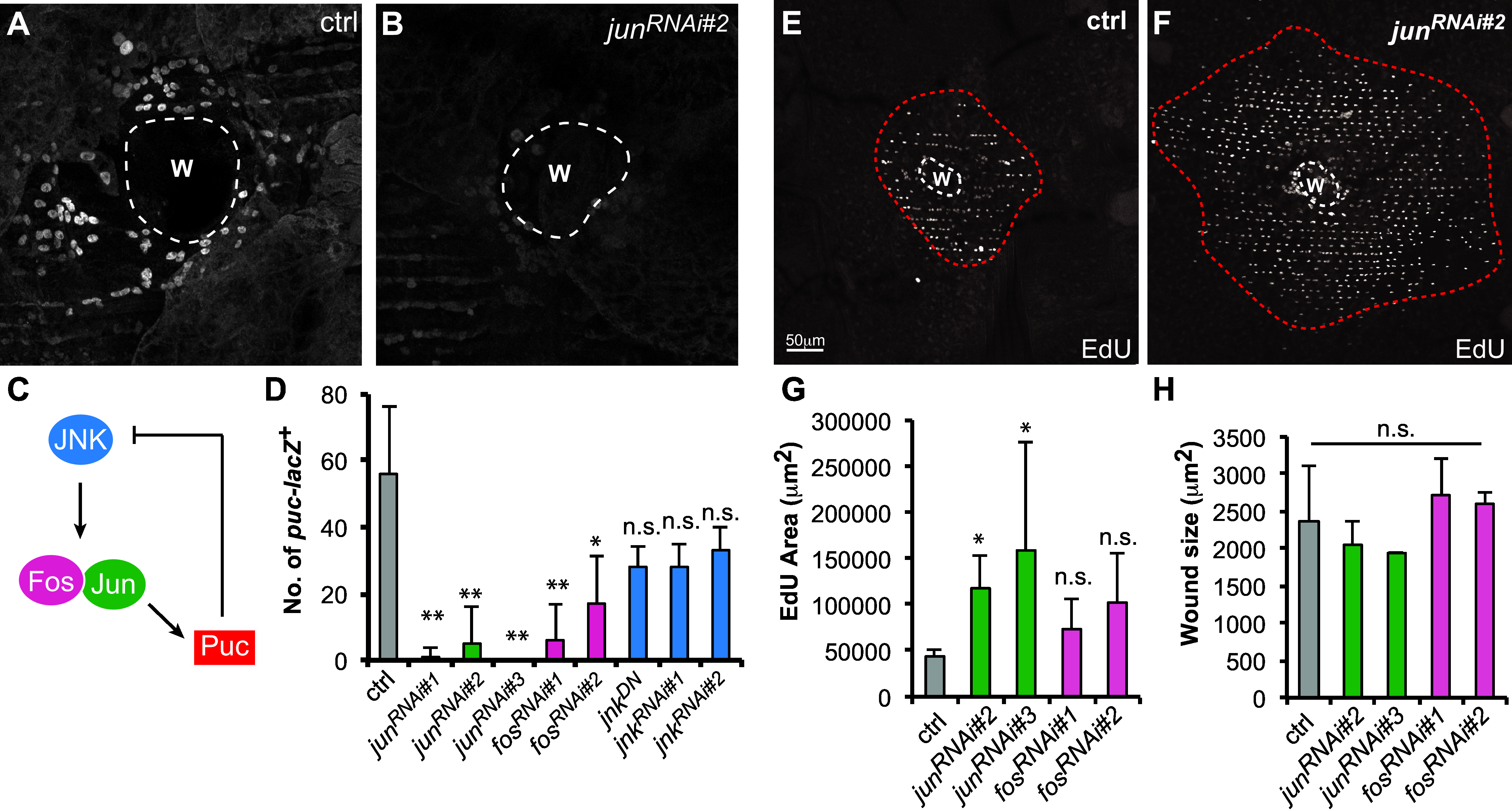

Supplement: S4 Fig — (A and B) Immunofluorescent images of the JNK reporter (puc-lacZ) expression around the fly wound at 2d post injury. (C) Diagram of core JNK signaling components. JNK (also known as Bsk in flies) activates the transcription factors Jun and Fos (making up the AP-1 complex) inducing Puc, a phosphatase, which feeds back to inhibit JNK. (D) Effectiveness of JNK pathway RNAi lines to inhibit the puc-lacZ expression. Shown is the average number of puc-lacZ+ nuclei at 2d. At least 10 flies were scored for each condition. (E and F) Immunofluorescent images of EdU staining in ctrl or epidermal specific jun knock down (junRNAi). EdU area is outlined (red dashed line). Wound scar (W, dashed white line). (G and H) Quantification of the AP-1 genes’ effect on the EdU response and wound size at 2d post injury. All transgenes were expressed with epidermal specific-Gal4 driver and examined. At least 3 flies were scored for each condition. Error bars represent standard deviation where ** p<0.01, * p<0.05 and n.s., not significant (p>0.05) are based on two-tailed Student's t test. (TIF) [file pone.0151251.s004.tif]

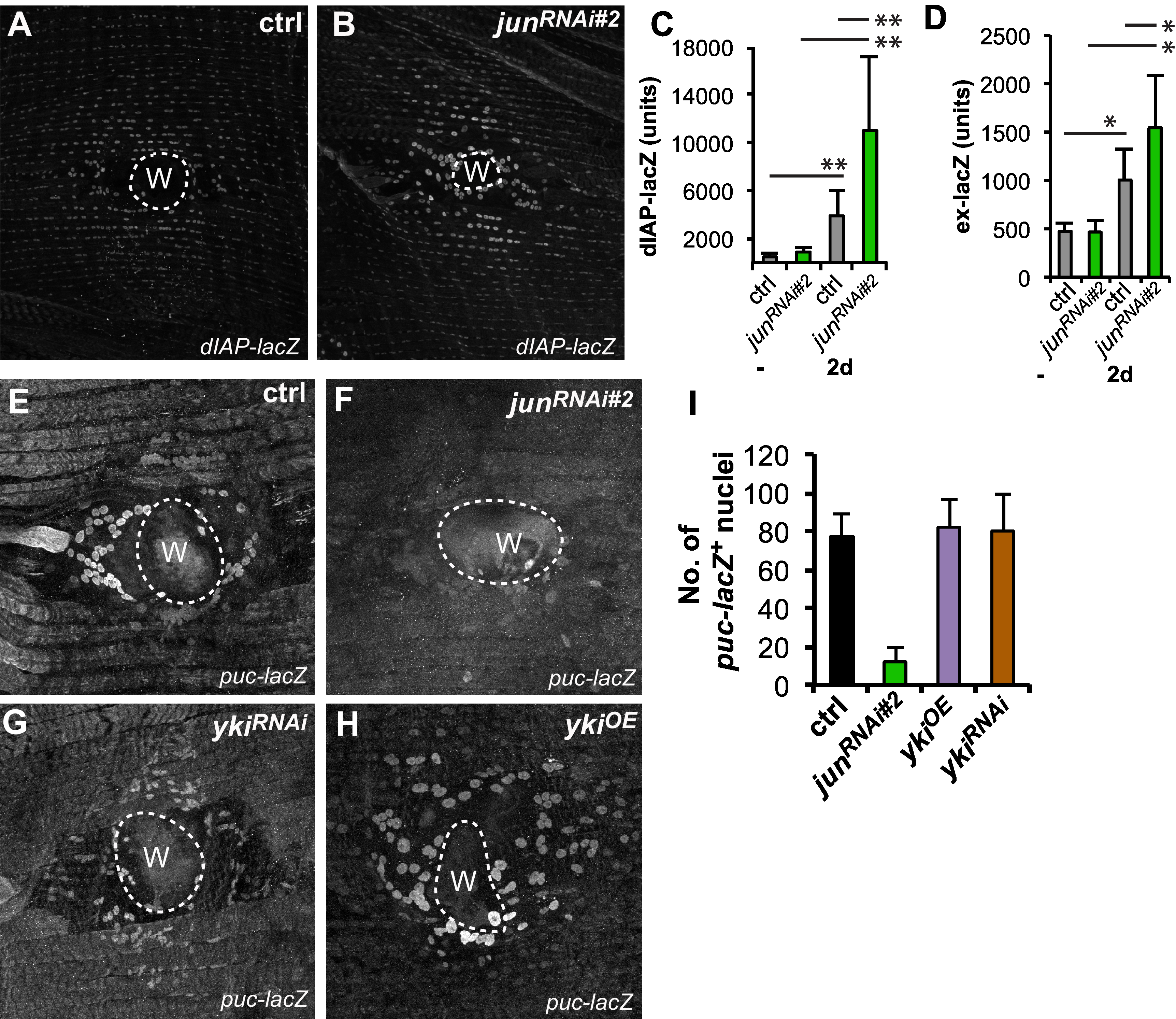

Supplement: S5 Fig — Knocking down jun in the adult fly epidermis enhances Yki dependent gene expression. Shown are representative immunofluorescent images of (A) ctrl and (B) junRNAi for the Yki reporter dIAP-lacZ at 2d post injury. (C) Quantification of Yki reporters dIAP-lacZ and (D) ex-lacZ expression in uninjured (-) or at 2d post injury (2d) in indicated conditions. (E-H) Yki signaling does not affect activation of the JNK reporter. Shown are representative immunofluorescent images of (E) ctrl, (F) junRNAi, (G) ykiRNAi, and (H) ykiOE for the JNK reporter, puc-lacZ, at 2d post injury. (I) Quantification of JNK reporters in indicated conditions. Wound scar is outlined (W, dashed white line). At least 3 flies were scored for each condition. Error bars represent standard deviation where *p<0.05, **p<0.01, and n.s., not significant (p>0.05) are based on two-tailed Student's t test. (TIF) [file pone.0151251.s005.tif]

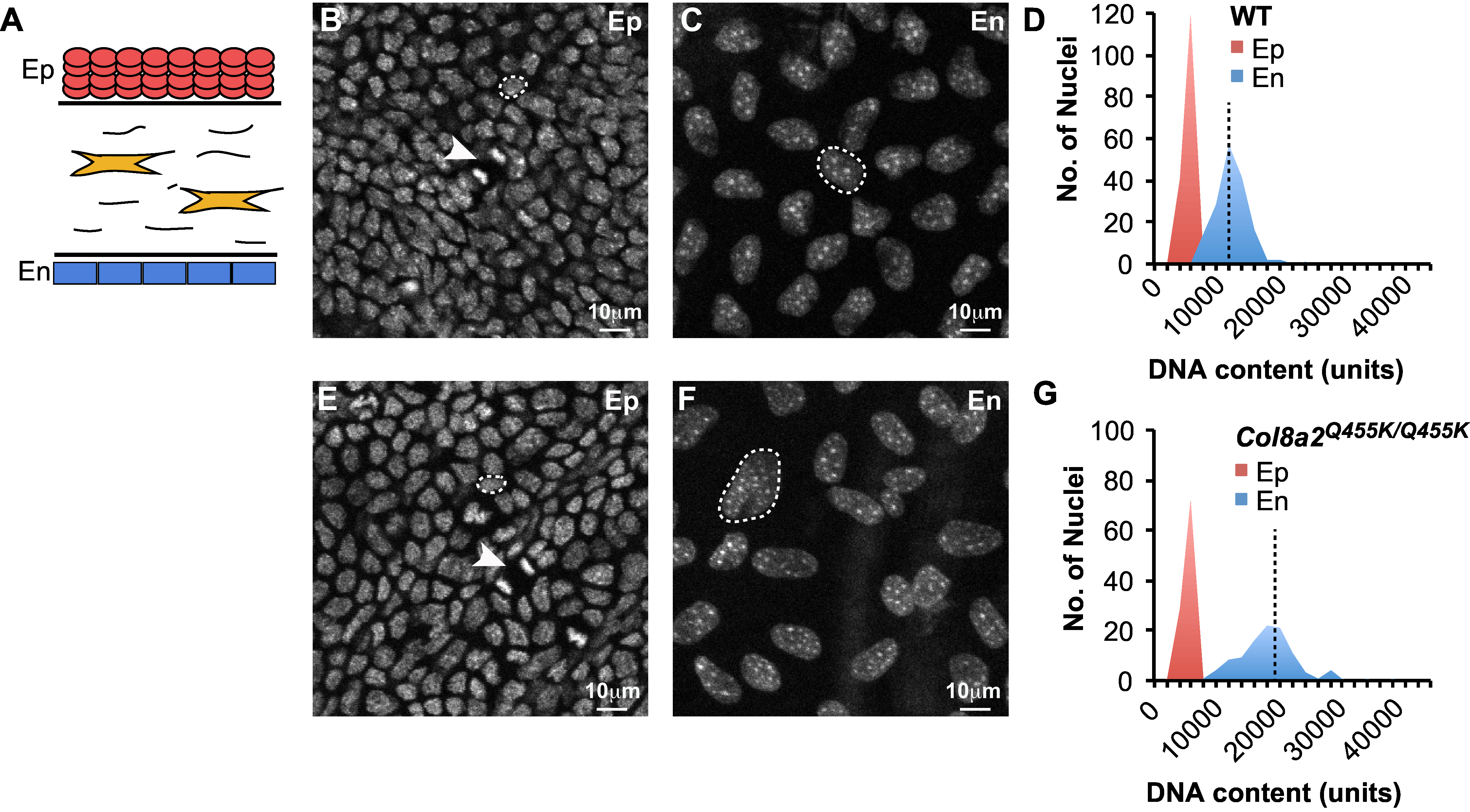

Supplement: S6 Fig — (A) A cross-section diagram of the cell types within mammalian cornea of the eye. The outer most layer is made of several layers of epithelial cells (Ep, red) and the inner most layer is a single row of endothelial cells (En, blue) separated by a stromal cell layer (yellow). (B, C, E and F) Immunofluorescent DAPI images of the (B and C) WT and (E and F) Col8a2Q455K/Q455K cornea Ep and En nuclei. Ep cells actively divide (arrowheads) and nuclei are small (example outlined) compared to differentiated En cell’s nuclei which are post-mitotic and larger. DNA content values were quantified from 3 representative endothelial (150,000μm2) regions from Ep vs En nuclei measured in (D) WT and (G) Col8a2Q455K/Q455K within corneas from age matched 7-month old male mice. (TIF) [file pone.0151251.s006.tif]
